# Cows' Milk and Consumption

**Published:** 1889-06-29

**Authors:** Francis Imlach

**Affiliations:** 30, Hope-street, Liverpool


### COW'S MILK AND CONSUMPTION.

SIR,—In your issue of June 8 there is a paragraph concerning the transmission of consumption from the cow to man by means of the milk, in which certain experiments of mine are not quite correctly described.

As the subject is one in which many of your readers will take a lively interest, I shall be glad if you can allow me to state the conclusions at which I have arrived.

It is an undoubted fact that consumption is common among cattle, and especially among milk cows in town byres. Even before Koch's well-known experiments on the transmission of tubercle, the hypothesis was put forth that consumption or phthisis in mankind was due to eating the flesh and drinking the milk of cows affected with this disease, and since 1884 it has been adopted with considerable confidence.

I spent a large part of 1883 in putting this supposition to the test. I bought consumptive or tuberculous cows, and fed young animals, such as calves, pigs, goats, guinea-pigs, and monkeys with their milk. But I never found that the disease had been conveyed to these young animals. Two monkeys died from tubercle, but these pets nearly always die of it in this climate, no matter how you feed them. I have, therefore, abandoned my belief that milk can convey this disease. If the contrary belief were to extend there would be no alternative but to give up cows' milk altogether. Fat and apparently healthy animals, yielding a plentiful suply of rich milk, may be in an early stage of the disease, and the better the breed the more likely is this to be the case. It is quite impossible to distinguish the affected animals at an early stage from those which are free from taint. Of course, the milk may be boiled. But does anyone boil children's milk? I have known it tried for a week or two, but no longer. If meat and milk really convey tubercle, which I now do not believe, I suggest that we eat mutton instead of beef, and look out for some substitute for milk. FRANCIS IMLACH,

30, Hope-street, Liverpool, June 18.

